# Increased Adhesion of *Listeria monocytogenes* Strains to Abiotic Surfaces under Cold Stress

**DOI:** 10.3389/fmicb.2017.02221

**Published:** 2017-11-14

**Authors:** Bo-Hyung Lee, Michel Hébraud, Thierry Bernardi

**Affiliations:** ^1^BioFilm Control, Biopôle Clermont Limagne, Saint-Beauzire, France; ^2^Université Clermont Auvergne, Clermont-Ferrand, France; ^3^Institut National de la Recherche Agronomique, Université Clermont Auvergne, UMR MEDiS, Saint-Genès-Champanelle, France

**Keywords:** *Listeria monocytogenes*, cold stress, adhesion, biofilm, BRT^®^, crystal violet staining, MATS, SEM

## Abstract

Food contamination by *Listeria monocytogenes* remains a major concern for some food processing chains, particularly for ready-to-eat foods, including processed foods. Bacterial adhesion on both biotic and abiotic surfaces is a source of contamination by pathogens that have become more tolerant or even persistent in food processing environments, including in the presence of adverse conditions such as cold and dehydration. The most distinct challenge that bacteria confront upon entry into food processing environments is the sudden downshift in temperature, and the resulting phenotypic effects are of interest. Crystal violet staining and the BioFilm Ring Test^®^ were applied to assess the adhesion and biofilm formation of 22 listerial strains from different serogroups and origins under cold-stressed and cold-adapted conditions. The physicochemical properties of the bacterial surface were studied using the microbial adhesion to solvent technique. Scanning electron microscopy was performed to visualize cell morphology and biofilm structure. The results showed that adhesion to stainless-steel and polystyrene was increased by cold stress, whereas cold-adapted cells remained primarily in planktonic form. Bacterial cell surfaces exhibited electron-donating properties regardless of incubation temperature and became more hydrophilic as temperature decreased from 37 to 4°C. Moreover, the adhesion of cells grown at 4°C correlated with affinity for ethyl acetate, indicating the role of cell surface properties in adhesion.

## Introduction

In recent decades, the foodborne pathogen *Listeria monocytogenes* has become a notable threat to food manufacturers, particularly those making ready-to-eat (RTE) foods (Jofré et al., 2016; Vongkamjan et al., 2016). Infection with this saprophytic and psychrotrophic gram-positive pathogen results in a high mortality rate, especially in susceptible groups such as pregnant women or senior populations (Orsi et al., 2011).

*Listeria monocytogenes* efficiently survives under extreme conditions, such as 40% w/v NaCl or pH 3.0 (Liu et al., 2005). The risk of listeriosis has increased with growing consumption of RTE foods or frozen foods requiring minimal heat treatment before consumption because food processing plants often utilize adverse conditions such as refrigeration, high salt concentration, or low pH to preserve foods. Moreover, *L. monocytogenes* persists by adhering to food contact surfaces, causing the contamination of final food products (Ferreira et al., 2014). A biofilm is a sessile community of bacterial cells embedded in a matrix of self-produced extracellular polymeric substances (EPS), including proteins, polysaccharides, and extracellular DNA. According to recent studies, the composition of EPS in the *L. monocytogenes* biofilm matrix is dominated by teichoic acids (Brauge et al., 2016; Colagiorgi et al., 2016). Biofilms are often multi-species in nature, and interactions with other bacteria may benefit biofilm formation by *L. monocytogenes* (Giaouris et al., 2015). Although maturity may vary depending on environmental conditions, fundamental biofilm growth involves bacterial adhesion to surfaces (Garrett et al., 2008).

Given increasing concerns that biofilms in food premises lead to food contamination with *L. monocytogenes*, studies have primarily compared the effects of temperature on biofilm formation by growing bacteria at different temperatures, most frequently ranging from 4 to 37°C, and demonstrated that bacteria survive and form biofilms at low temperatures (Di Bonaventura et al., 2008; Nilsson et al., 2011). In general, total biomass production by *L. monocytogenes* strains is augmented with increased incubation temperature, regardless of the adhesion surface, including hydrophilic stainless-steel coupons and hydrophobic polystyrene culture plates. One study showed that storage of *L. monocytogenes* strains at –20°C for 6 and 24 months increased adhesion and biofilm formation on various surfaces, including polystyrene microtiter plates and stainless-steel (Slama et al., 2012). Given its exceptional adaptive ability to mitigate and survive harsh environments, biofilm formation by *L. monocytogenes* may be an adaptation response to stress (Tasara and Stephan, 2006). However, direct observation of cold stress-induced biofilm production by *L. monocytogenes* has not been reported to date.

In this study, a total of 22 *L. monocytogenes* strains of diverse origins and serogroups were investigated to elucidate the impact of cold on phenotypic changes. Cells acclimatized at 37 and 4°C were exposed to cold to evaluate the effects of cold stress on bacterial adhesion and biofilm formation on polystyrene and stainless-steel surfaces using the BioFilm Ring Test^®^ (BRT^®^), crystal violet (CV) staining, and scanning electron microscopy (SEM). Furthermore, microbial affinity to solvents (MATS) analysis was performed to assess cell surface physicochemical properties and their relationship to surface adhesion characteristics.

## Materials and Methods

### *Listeria monocytogenes* Isolates and Culture Conditions

A panel of 22 isolates of *L. monocytogenes* from human listeriosis cases, animals, foods and food-related premises were used in this study (**Table 1**). All strains were analyzed by the Institut Pasteur (Paris, France) for serogrouping using a multiplex PCR assay (Doumith et al., 2004). Serogroup IVb includes serovars 4b, 4d, and 4e; serogroup IIb includes serovars 1/2b, 3b and 7; serogroup IIa includes 1/2a and 3a; and serogroup IIc includes serovars 1/2c and 3c.

**Table 1:** *Listeria monocytogenes* strains used in this study.

Strain	Lineage	Serogroup	Origin	Reference
1	I	IVb	Human, epidemic (pasteurized milk)	ScottA
2	I	IVb	Human, epidemic (hot dog)	
3	I	IVb	Meat (sausage)	
4	I	IVb	FCS^a^ in FPE^b^	
5	I	IIb	Cow	
6	I	IIb	Human, sporadic	
7	I	IIb	Chocolate milk, epidemic	
8	I	IIb	FCS in FPE	
9	I	IIb	Lean meat	
10	II	IIa	Not known	
11	II	IIa	Cow	
12	II	IIa	Hot dog, sporadic	
13	II	IIa	Human, sporadic (hot dog)	
14	II	IIa	Meat (batter)	
15	II	IIa	Meat (sausage)	
16	II	IIa	FCS in FPE	
17	II	IIa	FCS in FPE	
18	II	IIa	Meat (cured ham)	
19	II	IIa	Meat (batter)	
20	II	IIc	Rabbit	EGDe
21	II	IIc	FCS in FPE	
22	II	IIc	Human	LO28

^a^*FCS: food contact surface ^b^FPE: food processing environment The contaminated food sources of certain epidemic and sporadic cases are specified*.

Strains were stored in Brain–Heart Infusion (BHI) broth (Laboratorios Conda, Spain) with 8.3% glycerol at –20°C, and each set of experiments was conducted with freshly recovered isolates on BHI agar (Laboratorios Conda, Spain). Strains were maintained on BHI agar for at least 2 days at 37°C by sub-culturing daily onto a fresh agar plate.

### Sample Preparation

**FIGURE 1:**
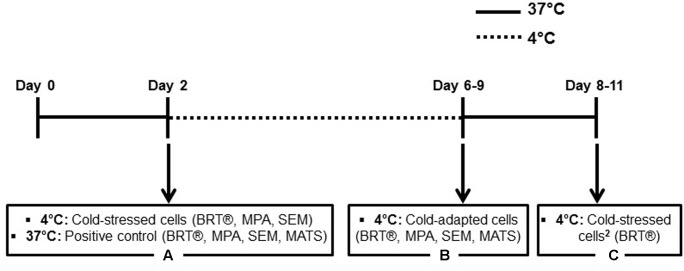
Experimental scheme. Time frame for incubation at 37 or 4°C is shown, with arrows indicating cells in stationary phase used for experiments. Each box (A–C) contains the temperatures and names used for sample conditions, with a list of experiments in parentheses.

Several colonies were harvested using a sterile inoculating loop, suspended in 20 ml of BHI broth and grown at 37°C with shaking at 100 rpm to reach stationary phase. After incubation for 15 h, stationary cells were pelleted by centrifugation at 5,000 × *g* for 10 min at room temperature, dispersed in 5 ml of fresh BHI broth by vortexing, and utilized for different experiments. Some cells were incubated at 37 and 4°C and were denoted positive control and cold-stressed cells, respectively (**Figure 1**, box A). A portion of the culture was diluted in 20 ml of BHI medium pre-cooled to 4°C to obtain an optical density at 600 nm (OD_600_) of 0.1 and brought to 4°C to grow under shaking at 100 rpm for 4 to 7 days until the cells reached stationary phase. Stationary cells were harvested by centrifugation at 5,000 × *g* for 10 min at 4°C and suspended by vortexing in fresh pre-cooled BHI broth for use as cold-adapted samples (**Figure 1**, box B). Some cold-adapted cells were streaked onto BHI agar with a sterile inoculating loop and incubated at 37°C overnight. This culture was then exposed to sudden cold stress as described above and designated cold-stressed cells^2^ (**Figure 1**, box C).

### Viable Cell Counts

Portions of the positive control (precultured at 37°C) and cold-adapted cells (precultured at 4°C) were used to obtain viable cell counts. After measuring OD_600_ with a spectrophotometer (Biomate3, Thermo Scientific, United States), 50 μl of culture was transferred to tryptone salt (TS) solution containing 0.1% w/v tryptone (Conda, Spain) and 0.85% w/v NaCl (Sigma–Aldrich, France) and 10-fold serially diluted in TS solution. From each dilution, 100 μl was spread on a BHI agar plate using sterile glass beads in triplicate. After overnight incubation at 37°C, colonies were counted to calculate colony forming units (CFU) per ml. Total CFU of 30 to 300 per plate were considered valid data.

Two experiments were performed for each condition.

### BRT^®^

The BRT^®^ assay was performed in a polystyrene 96-well microplate (BRT kit C004, BioFilm Control, France) as described by Chavant et al. (2007).

A freshly prepared culture was measured at OD_600_ to obtain a final OD_600_ of 0.5 (approximately 1.2 × 10^9^ CFU/ml) in BHI broth kept at two temperatures: 4°C for cold-stressed and cold-adapted conditions, and room temperature for a positive control. A portion of the suspension was used to perform a threefold dilution to obtain an OD_600_ of 0.17 (approximately 3.8 × 10^8^ CFU/ml). Toner 4 (magnetic beads) was added to a final concentration of 10 μl ml^-1^. After homogenization of these mixtures, 200 μl of each bacterial suspension was deposited in each well of the microplate in triplicate. Wells containing only BHI broth with magnetic beads were added as negative controls.

Microplates were kept static for 24 h at 4°C for cold-stressed (precultured at 37°C) and cold-adapted (precultured at 4°C) conditions and at 37°C for the positive control. After incubation, wells were covered with a few drops of Liquid Contrast (inert and non-toxic oil), and the plates were placed on a magnetic block with 96 mini-magnets centered under the 96 wells of the microplate for 1 min to apply magnetic fields that attracted mobile beads, creating a quantifiable spot above each mini-magnet. The bottoms of the plates were scanned with a plate reader and analyzed using BFC elements 3^®^ software (BioFilm Control, France) to obtain a numerical value termed the BioFilm Index (BFI) for each well, which ranged from 0 to 20 depending on the size and intensity of the spot. A BFI of approximately 20 corresponded to high magnetic bead mobility, implying no or very few sessile cells, while a lower BFI or a value of zero resulted from the immobilization of beads by sessile cells.

At least four experiments were performed, with triplicate wells for each condition.

### Microtiter Plate Assay (MPA)

The procedure to prepare a 96-well microplate for microtiter plate assay (MPA) was the same as that described above (BRT^®^), except that no Toner 4 was added. The assay was performed as previously described by Doijad et al. (2015), with slight modifications. After static incubation for 24 h, the absorbance values of negative controls and total cell densities, including sessile and planktonic cells, were measured using a microplate reader (EL800, BioTek, United States) at OD_600_.

Plates were inverted, and the media and planktonic cells were discarded via gentle tapping. To remove loosely attached bacteria, wells were washed twice with 300 μl of sterile saline solution (8.5 g of NaCl per l). Sessile cells were fixed with 300 μl of 96% ethanol (Sigma–Aldrich, France) for 20 min and air-dried for 2–3 h at room temperature after removal of the ethanol until no standing moisture was visible. To stain the bacterial biomass, a 0.1% w/v CV (Merck KGaA, Germany) solution was filtered (0.22-μm filter, Millipore, France), and 220 μl was added to each well. After static incubation for 30 min, CV solution was removed by sharply flicking the plates while upside down. Wells were washed three times with 300 μl of saline, followed by tapping them upside down on paper towels. Plates were air-dried for 3–4 h, filled with 150 μl of 33% v/v acetic acid (Sigma–Aldrich, France) and placed on a plate shaker with slight agitation for 10 min to completely destain CV and obtain a homogenized solution. Destained CV levels were determined using a microplate reader at OD_600_.

At least four experiments were performed, with triplicate wells for each condition.

### Physicochemical Experiments

The MATS partitioning method (Bellon-Fontaine et al., 1996) was performed to define bacterial cell surface properties. This method involves comparing the affinities of microbial cells for pairs of monopolar and nonpolar solvents, which have similar van der Waals surface tension components. In this study, the following sets of solvents were used: (i) chloroform, an acidic solvent (electron acceptor), and hexadecane, a nonpolar *n*-alkane, and (ii) ethyl acetate, a basic solvent (strong electron donor), and decane, a nonpolar *n*-alkane (Sigma–Aldrich, France).

Cultures grown until stationary phase at 37 and 4°C were pelleted by centrifugation at 5,000 × g for 10 min at room temperature and 4°C, respectively. Sterile 0.15 M NaCl (Sigma–Aldrich, France) solutions pre-incubated at 37 and 4°C were used to wash pellets, in compliance with the original culture temperatures, followed by centrifugation. Bacterial suspensions were prepared to obtain an OD at 400 nm (OD_400_) of approximately 0.6 to 0.7, and the initial OD_400_ was measured as [A_0_]. A suspension in a volume of 2.4 ml was vortexed for 60 s with 0.4 ml of each solvent in a glass tube. The mixture was allowed to stand static for 15 min to ensure the complete separation of both phases. The absorbance of the aqueous phase was measured at OD_400_ [A]. The percentage of cells in each solvent was calculated using the following equation: percent affinity = [1–(A/A_0_)] × 100.

Each experiment was performed in quadruplicate with independently grown bacterial cultures.

### SEM

Biofilms were grown on sterile stainless-steel coupons to visualize adhesion patterns, biofilm architecture, and cell morphologies via SEM with positive control, cold-stressed, and cold-adapted cells.

Fresh cultures in BHI broth were prepared to obtain an OD_600_ of 0.5, and 7 ml of each bacterial suspension was poured into a petri dish (55-mm diameter) containing a sterile stainless-steel coupon (AISI 304, mean roughness = 0.064) and statically incubated for 24 h at 37°C for a positive control and at 4°C for the cold-stressed and cold-adapted conditions. After removing the cultures using a pipette, the coupons were gently washed twice by filling the petri dishes with sterile saline solution to remove nonadherent cells. Sessile cells and biofilms were fixed on each coupon in 10 ml of a solution containing 3% glutaraldehyde in 0.2 M cacodylate buffer (pH 7.4) in a 50-ml glass beaker at 4°C for a minimum of 1 h to overnight. Coupons were washed three times for 15 min each via immersion in cacodylate buffer, followed by dehydration using a graded ethanol series (70, 90, and 100%) three times for 15 min each. Further dehydration was performed in a 50:50 mixture of ethanol:hexamethyldisilazane (HMDS) three times for 10 min each. Samples were immersed in pure HMDS (Delta Microscopies, France) twice for 10 min, followed by air-drying overnight at room temperature. Coupons were mounted on stubs using adhesive carbon tabs, sputter-coated with gold-palladium (JFC-1300, JEOL, Japan) and observed with a scanning electron microscope (JEOL 6060-LV, JEOL, Japan) at 5 kV in high-vacuum mode.

### Statistical Analysis

A *t*-test was performed on data comparing cold-stressed and cold-adapted cells or positive control and cold-adapted cells to test for statistically significant differences. Correlations were evaluated to identify any effects of cell surface properties on bacterial adhesion and biofilm formation by calculating the Pearson’s correlation coefficient. All data were analyzed using Prism 7 software (Graphpad software Inc., United States), and significance was assigned at *p* < 0.05.

## Results

### Viable Cell Counts

Six stains composed of four different serogroups with diverse origins were selected for viable cell count tests to verify the relationship between OD_600_ values and viable cell numbers. As shown in **Table 2**, the two stationary cultures acclimated to 37 and 4°C resulted in comparable numbers of viable cells, with no significant differences.

**Table 2:** Viable cell counts of six strains grown at stationary phase at two temperatures.

Strain	37°C	4°C
1	9.36 ± 0.03	9.46±0.05
4	9.33 ± 0.03	9.50±0.05
6	9.30 ± 0.03	9.45±0.03
11	9.27 ± 0.06	9.43±0.01
14	9.24 ± 0.01	9.33±0.02
20	9.22 ± 0.04	9.35±0.20

*Data are presented as the mean log CFU/ml ± standard deviation at an OD_600_ of 1*.

### Evaluation of Adhesion and Biofilm Formation on a Polystyrene Surface

#### BRT^®^

**FIGURE 2:**
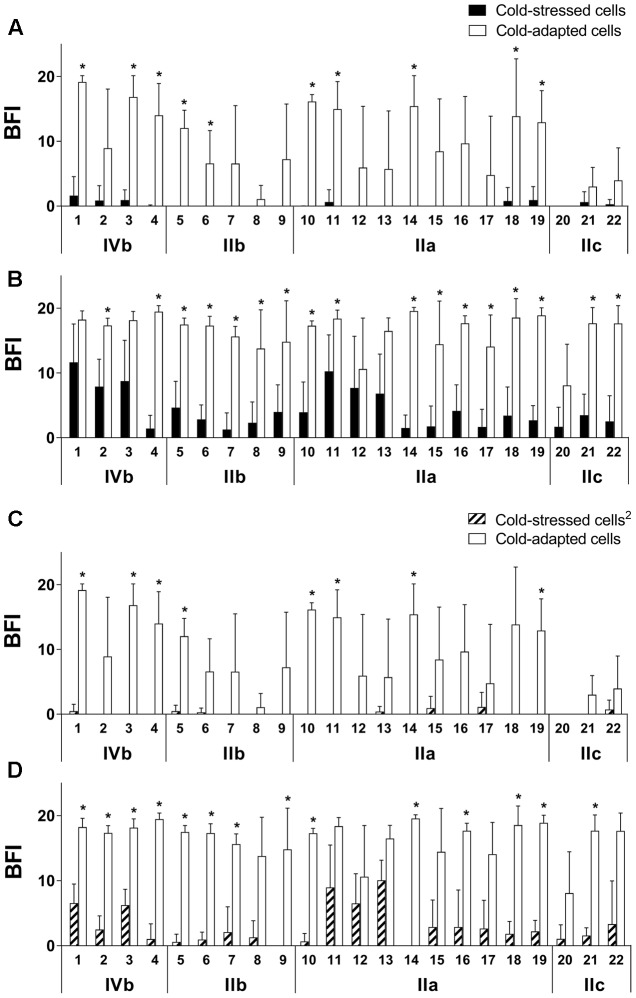
Increased adhesion of cold-stressed cells compared to cold-adapted cells, measured by BRT^®^. Sudden exposure to cold for the first time was denoted as cold-stressed cells **(A,B)**, and exposure for a second time was denoted as cold-stressed cells^2^ **(C,D)**; initial inocula were at an OD_600_ of 0.5 **(A,C)** and 0.17 **(B,D)**. Strains and serogroups are indicated on the X-axis, and data are presented as the mean ± standard deviation of the BFI. A BFI of 0 represents full blockage of the magnetic beads. ^∗^*p* < 0.05.

The ability of 22 *L. monocytogenes* strains to adhere to an abiotic surface was analyzed using BRT^®^. BFI, with a value ranging from 20 to 0, is associated with the extent of blockage of magnetic beads (Toner 4) by sessile bacterial cells at the bottom of polystyrene microplate wells. Therefore, differences in BFI are caused by varying abilities to adhere. All strains exhibited higher adhesion ability when exposed to cold shock by demonstrating lower BFI scores (**Figure 2**). Statistical differences (*p* < 0.05) in BFI values between cold-stressed and cold-adapted cells were observed for 19 strains in either of the two inocula (**Figures 2A,B**). Further tests were performed to verify whether this adhesion phenomenon was reversible. Cold-adapted cells were cultured at 37°C and re-exposed to a sudden temperature downshift (cold-stressed cells^2^). Interestingly, the same response was observed for adhesion profiles (**Figures 2C,D**). No significant difference was observed for any strain between cells that were exposed to sudden cold shock for the first time (cold-stressed cells) and the second time (cold-stressed cells^2^), demonstrating that enhanced adhesion upon cold exposure represents a transient phenotype switch (Supplementary Figure 1).

When positive control cells were incubated at 37°C, all strains completely blocked the beads, resulting in a BFI of 0 for all inocula (data not shown).

#### MPA

Microtiter plate assay was performed to assess sessile biomasses and total cell densities after incubation for 24 h at 4°C for cold-stressed and cold-adapted conditions and at 37°C for positive control. Much greater biomass quantities were obtained by CV staining for the positive control than for cold-stressed or cold-adapted cells (Supplementary Figure 2).

**FIGURE 3:**
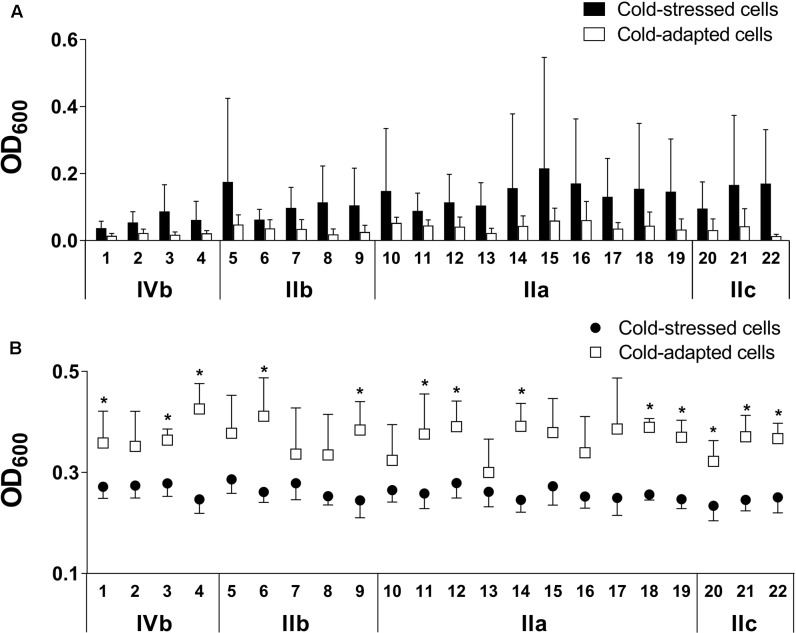
Cold-stressed cells form more biomass than cold-adapted cells **(A)**, while cold-adapted cells exceed total cell densities **(B)** as assessed using microtiter plate assay (MPA). Adherent cells are quantified by CV staining **(A)**, and total cell densities combining planktonic and sessile cells are measured based on the turbidity of wells **(B)**. Strains and serogroups are indicated on the X-axis, and data are presented as the mean ± standard deviation. ^∗^*p* < 0.05.

Cold-adapted cells showed overall higher total cell densities than cold-stressed cells; 13 of the 22 strains were statistically significant (*p* < 0.05, **Figure 3B**). Cold-stressed cells, however, resulted in more sessile bacterial communities than cold-adapted cells (no significance found) as quantified by CV staining (**Figure 3A**). The higher total cell densities obtained for cold-adapted cells were primarily attributable to planktonic cells. Based on these findings, increased cell numbers in cultures did not result in bacterial adhesion, indicating that enhanced adhesion is a distinct feature of cold-stressed cells.

### Cell Surface Physicochemical Properties

Positive control and cold-adapted cells, grown at 37 and 4°C until stationary phase, respectively, were prepared for the MATS test to compare the surface physicochemical properties of cells that acclimated to different temperatures. The MATS results obtained for all 22 *L. monocytogenes* grown at 37 and 4°C until stationary phase in BHI media are shown in Supplementary Figure 3.

**FIGURE 4:**
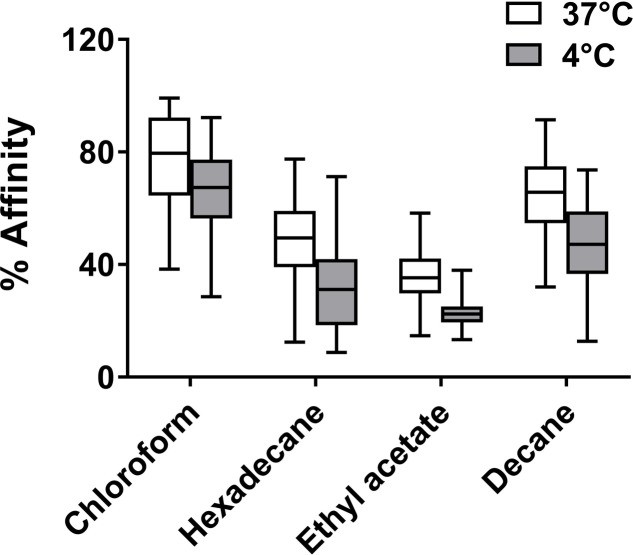
Solvent affinities (%) of all 22 *Listeria monocytogenes* strains in stationary phase at 37 and 4°C. Box plot whiskers indicate minimum and maximum values, and the line in the middle of the box is plotted at the median. The affinity for each solvent under two temperatures differs significantly (*p* < 0.01).

As shown in **Figure 4**, the general affinity of the 22 *L. monocytogenes* for chloroform (an electron-acceptor solvent) was higher than the affinity for hexadecane (a non-polar solvent), regardless of the culture temperature, indicating the strong electron donor nature of these bacteria. Likewise, bacterial affinity for ethyl acetate (an electron-donating solvent) was lower than for decane (a nonpolar solvent), indicating that the electron-accepting nature of the bacteria grown at either temperature was weak. Affinity for hexadecane decreased from 49 ± 14% at 37°C to 32 ± 15% at 4°C (*p* < 0.01), demonstrating that cell surfaces became relatively more hydrophilic as cells adapted to cold temperature.

**FIGURE 5:**
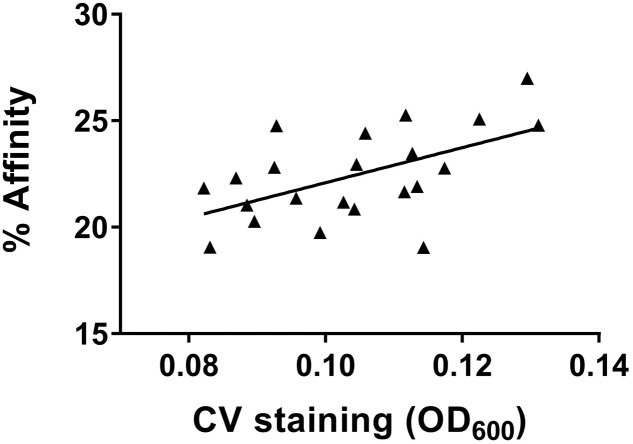
Correlation (*r*^2^ = 0.3055, *p* < 0.01) between affinity for ethyl acetate and quantification of adherent cells, measured by CV staining of cold-adapted cells.

Relationships between the affinities for the four solvents and adhesion data obtained via two methods, CV staining and BRT, were evaluated using Pearson’s correlation coefficient (*r*^2^). A positive correlation was identified between the adhesion results obtained from CV staining and the affinity for ethyl acetate obtained from the MATS test for cold-adapted cells grown at 4°C (*r*^2^ = 0.3055, *p* < 0.01) (**Figure 5**).

### SEM Observation

**FIGURE 6:**
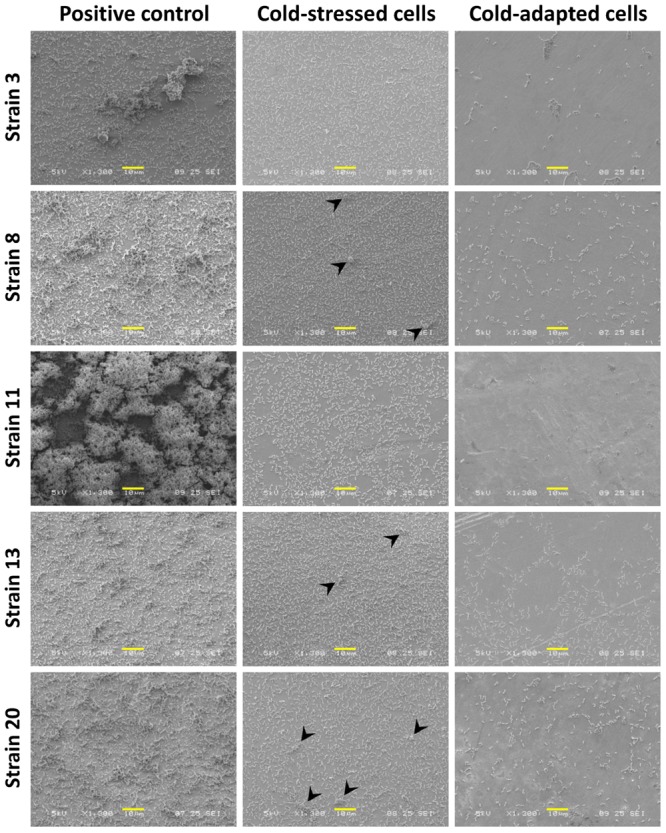
Comparison of *L. monocytogenes* biofilm formation under different conditions. Positive control cells demonstrated great variance in terms of interstrain biofilm formation (left column), while cold-stressed cells formed a single biofilm layer with cell aggregates (arrowhead) (middle column), and cold-adapted cells adhered sparsely on the surface (right column). Scale bars: 10 μm.

**FIGURE 7:**
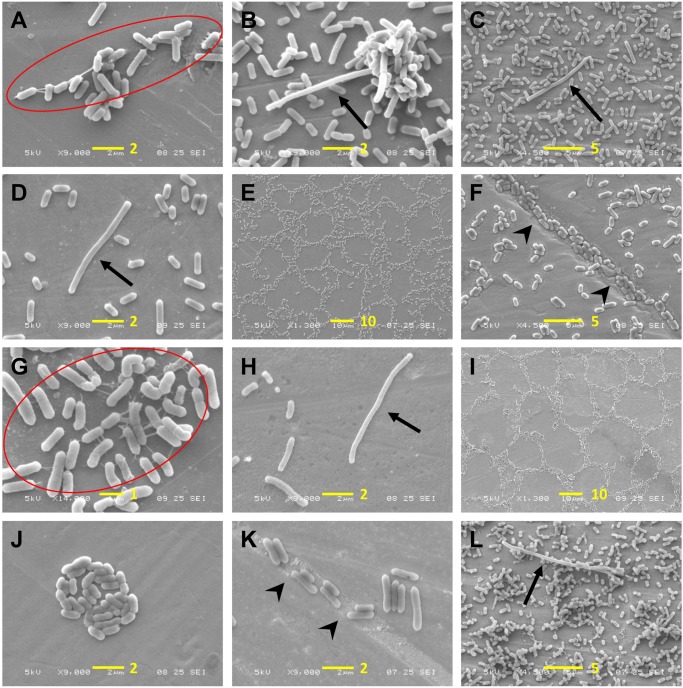
Observation of *L. monocytogenes* adhesion patterns and cellular morphology. **(A–F)** Cold-stressed cells; **(G–K)** cold-adapted cells; **(L)** positive control cells. EPS are marked in red circles, arrows indicate elongated cells and arrowheads indicate cells in crevices. A scale bar (length in μm) is indicated in yellow in each figure.

Scanning electron microscopy images of cells grown under all three tested conditions (positive control, cold-stressed, and cold-adapted cells) were obtained to analyze surface colonization patterns and biofilm structures as well as the morphologies of individual cells. Low to high magnifications were applied over several zones. There was greater variance in the maturity of biofilms among strains grown under positive control conditions (**Figure 6**, left column), showing that the biofilm-forming capability of *L. monocytogenes* is strain-dependent. Conversely, under cold-stressed and cold-adapted conditions, the variance among strains was less obvious, primarily because no homogeneous mature biofilms were produced. However, cold-stressed cells underwent surface colonization with cell aggregates (arrowhead) resulting from sessile cell division (**Figure 6**, middle column), while analysis of cold-adapted cells revealed the attachment of individual cells in the absence of noticeable cell clusters (**Figure 6**, right column). Extracellular matrix was observed at high magnification (X 9,000 and higher) among individual cells and between cells and the stainless-steel surface (**Figure 7**, red circle). Irregular cell sizes were observed under all conditions, but significant cell elongation was more frequently noted among cold-stressed and cold-adapted cells (**Figure 7**, arrow). This result may be because positive control cells formed more complex biofilm structures that limited the distinction of individual cell morphologies. Similar to a previous report by Harvey et al., spatial colonization was observed, constituting a network of microcolonies (**Figures 7E,I**) (Harvey et al., 2007). Cells were often found in indented substrate surfaces resulting from scratches on the coupons (**Figure 7**, arrowhead).

## Discussion

Certain bacteria adapt to inhabit environments by assuming different forms that are favorable to survival, such as planktonic cells, sessile biofilm communities or spore formation. Biofilms of *L. monocytogenes* in food processing environments are of great concern for food contamination. *L. monocytogenes* adapts to the harsh environments employed by food processing facilities, such as antibacterial agents or refrigeration, and reports have demonstrated that exposing bacteria to sublethal stress leads to cross-protection or cross-adaptation to various stresses and lethal factors (Lou and Yousef, 1997; Lundén et al., 2003). Biofilm production by *L. monocytogenes* is stimulated to protect against various stressful conditions, making bacterial elimination a serious challenge at food processing facilities (Giaouris et al., 2014).

Significant variation in biofilm production under various conditions was observed for one *L. monocytogenes* strain, indicating that intra-strain phenotype changes are dependent on experimental settings (Nowak et al., 2015). Moreover, the interstrain variability of biofilm formation has been extensively studied with a focus on its relationships to serogroups or persistence in the food industry. However, a study employing 143 *L. monocytogenes* strains indicated that experimental settings such as temperature and culture media affect the comprehension of biofilm formation and its relationship to serotype or origin (Kadam et al., 2013). Recently, a study of 98 *L. monocytogenes* strains revealed no correlation between serological groups and biofilm production (Doijad et al., 2015).

Numerous methods and devices have been developed to detect or quantify biofilms, including staining-based quantification methods, visual identification by microscopy, viable and culturable cell counts, and devices to test bacterial adhesion (Azeredo et al., 2017). BRT^®^ is a microbial adhesion test that is primarily used to assess the simultaneous phenotype switch from planktonic cells into sessile cells. In BRT^®^, microorganisms are added to microplate wells in planktonic form, some of which adhere to the bottoms of the wells during incubation and switch to a sessile form that hinders the magnetic beads attracted to the magnetic block. Results vary with experimental conditions that affect the process, including planktonic cell growth and adhesion to polystyrene microplates, as well as sessile cell growth. Recently, a new approach to BRT^®^, designated cBRT^®^, was developed using serial 10-fold dilutions of bacterial suspensions to better discriminate biofilm-forming abilities among strains (Di Domenico et al., 2016). However, in the current study, all 22 *L. monocytogenes* strains revealed homogenous adhesion behavior that was not discernible by cBRT^®^ under the same experimental conditions, i.e., cold-adapted or cold-stressed conditions or incubation at 37°C. Nevertheless, cBRT^®^ was sensitive for discriminating between cold-stressed and cold-adapted cells in terms of bacterial adhesion.

Cells undergo cold shock when subjected to a sudden downshift in temperature. Such a rapid environmental change induces modifications in bacterial cell surface proteins and lipid composition to maintain membrane fluidity homeostasis, which presumably facilitates adhesion as an adaptation strategy against adverse conditions. This behavior may be advantageous for bacterial survival in FPEs where cells might be exposed to sudden cold shock. The current study employed preculture temperatures of 37 and 4°C to compare differences in adhesion characteristics upon further exposure to 4°C. BRT^®^ and MPA results revealed that cold-stressed cells (precultured at 37°C) are more efficient at forming biofilms, while cold-adapted cells (precultured at 4°C) favor growth in the planktonic state. *L. monocytogenes* cells entering the food processing chain are exposed to temperature downshifts, such as ambient temperature in outdoor food materials or optimal temperature in infected animals to refrigeration temperatures used during food processing or storage. When introduced to the food processing chain, *L. monocytogenes* adhesion to food contact surfaces is potentially fortified by cold shock, which will increase the chance of food product contamination. Once adapted to the cold, the bacteria in final food products will proliferate to hazardous levels during distribution and storage.

The heterogeneity of a population contributes to the adaptation of *L. monocytogenes* to sublethal conditions, accompanied by phenotypic and genetic changes (Abee et al., 2016). When cold-adapted cells were returned to 37°C and re-exposed to cold, they exhibited the same enhanced adhesion, which was indistinguishable by BRT^®^ (Supplementary Figure 1), demonstrating that this transient trait was reacquired when cold stress was removed.

All 22 strains retained basic (electron-donating) properties (a higher affinity for chloroform than hexadecane), regardless of growth temperature, and became more hydrophilic with decreased temperature, as previously described (Chavant et al., 2002). The adhesion data obtained from CV staining of all 22 *L. monocytogenes* correlated best with cell affinity for ethyl acetate under cold-adapted conditions (**Figure 5**). This finding aligned with that of Briandet et al., who observed a linear correlation between listerial adhesion to stainless-steel and an affinity for ethyl acetate at different temperatures (37, 20, 15, and 8°C) in the presence of NaCl (Briandet et al., 1999). Physicochemical properties, including the hydrophobicity of bacterial cells versus that of a substratum, affect the interfacial interactions involved in bacterial attachment to abiotic surfaces. Nonetheless, this trait is negligible in the context of building a mature biofilm structure that is highly dependent on bacterial growth kinetics and EPS production, thus explaining the absence of an obvious relationship between cellular surface properties and biofilm formation at 37°C in the current study and in the literature (Cunliffe et al., 1999; Chmielewski and Frank, 2003).

Scanning electron microscopy observations supported the quantitative results obtained with CV staining and BRT^®^. **Figure 6** shows variable biofilm maturity among the strains. This variability may be attributable to differential biofilm production capabilities, although divergent biofilm kinetics among strains are unable to be excluded. Some strains may have already begun to disperse, while others were still in the process of structuring mature biofilms. SEM observation confirmed the higher bacterial adhesion of cold-stressed cells, along with the formation of cellular aggregates, while cold-adapted cells were only able to form a single sparse layer of adherent cells. This finding aligns with the BRT^®^ results, demonstrating that BRT^®^ is applicable for testing the early step of biofilm formation. Moreover, our observations of cells that densely accumulated in the crevices and scratches of stainless-steel surfaces strongly support the thorough cleaning of food contact surfaces to eliminate bacteria, although this may also further damage surfaces and create additional niches for bacterial adhesion.

In the current study, enhanced adhesion of sudden cold-stressed *L. monocytogenes* cells was observed for the first time. CV staining and SEM observation revealed that *L. monocytogenes* possesses dramatic interstrain variances in biofilm production, independent of origin or serotype. BRT^®^ is shown to be a sensitive tool to discern the first layer of biofilm formation, facilitating the detection of increased adhesion of cold-stressed cells in the current study. Interestingly, the adhesion of cold-adapted cells correlated with an affinity for ethyl acetate. Further study of cold-stressed cells during cross-adaptation to other stress factors, such as dehydration or antimicrobial agents, will add to our understanding of the behavior of *L. monocytogenes* in the food processing industry.

## Author Contributions

B-HL conceived, designed, and conducted experiments, analyzed the results, and drafted the manuscript. All authors contributed to the experimental design and reviewed and approved the final manuscript.

## Conflict of Interest Statement

The authors declare that the research was conducted in the absence of any commercial or financial relationships that could be construed as a potential conflict of interest.
