# Supplementary material for: Increased Adhesion of Listeria monocytogenes Strains to Abiotic Surfaces under Cold Stress
Source: Front Microbiol. 2017 Nov 14;8:2221. doi: 10.3389/fmicb.2017.02221 (PMC5695204; doi:10.3389/fmicb.2017.02221)
Supplement: Supplementary file 1 [file Data_Sheet_1.pdf]

Supplementary Material

Increased adhesion of *Listeria monocytogenes* strains to abiotic surfaces under cold stress

Bo-Hyung Lee*, Michel Hébraud, Thierry Bernardi

*** Correspondence:** Bo-Hyung LEE, 2bohyung@gmail.com

Supplementary Figures

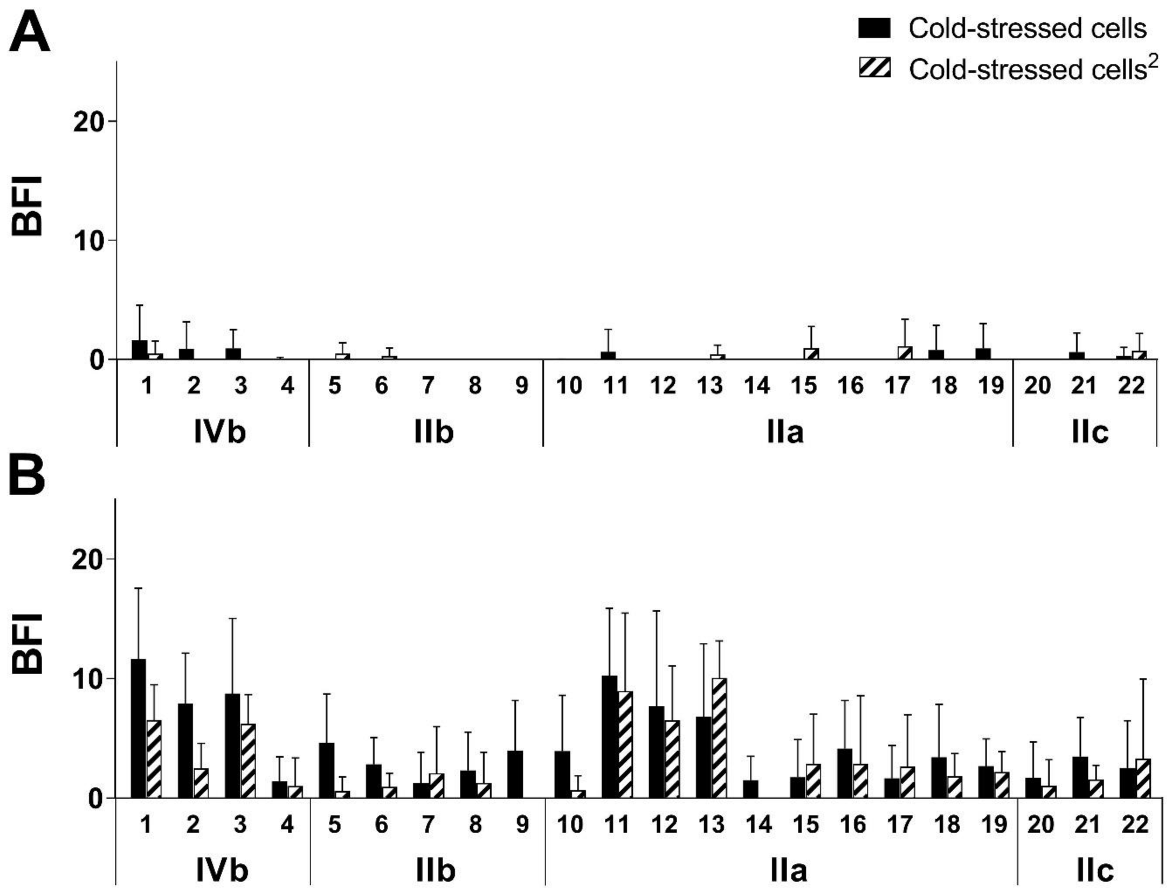

Supplementary Figure 1. Recovery of cold shock-induced adhesion is demonstrated by BRT®. Cold-stressed cells (exposed to cold shock for the first time) and cold-stressed cells^2 (exposed to cold shock for the second time) exhibit indistinguishable adhesion profiles. Initial inocula at OD_{600} of 0.5 (A) and 0.17 (B). Data are presented as the mean ± standard deviation.

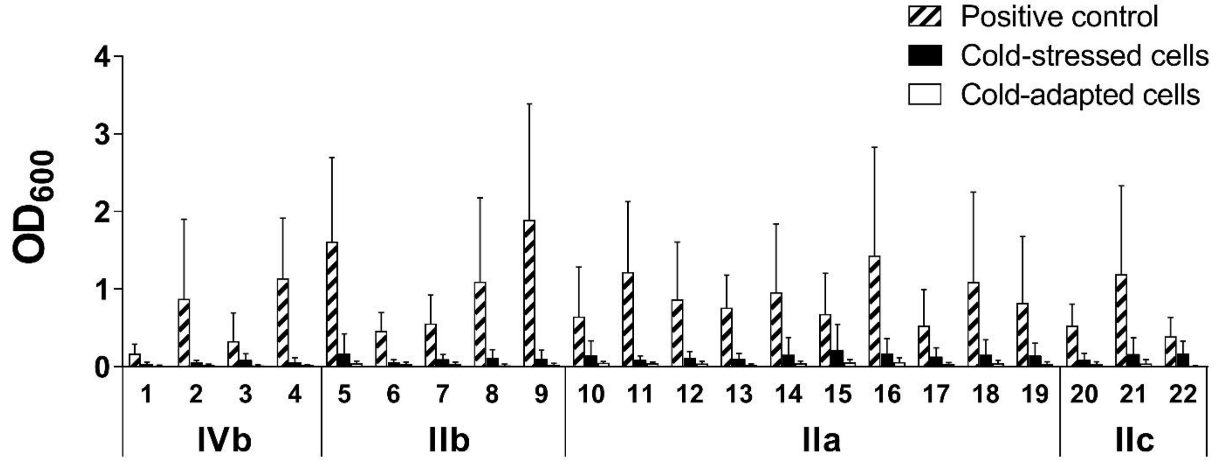

Supplementary Figure 2. Comparison of total biomass measured by CV staining. Data are shown as the mean ± standard deviation.

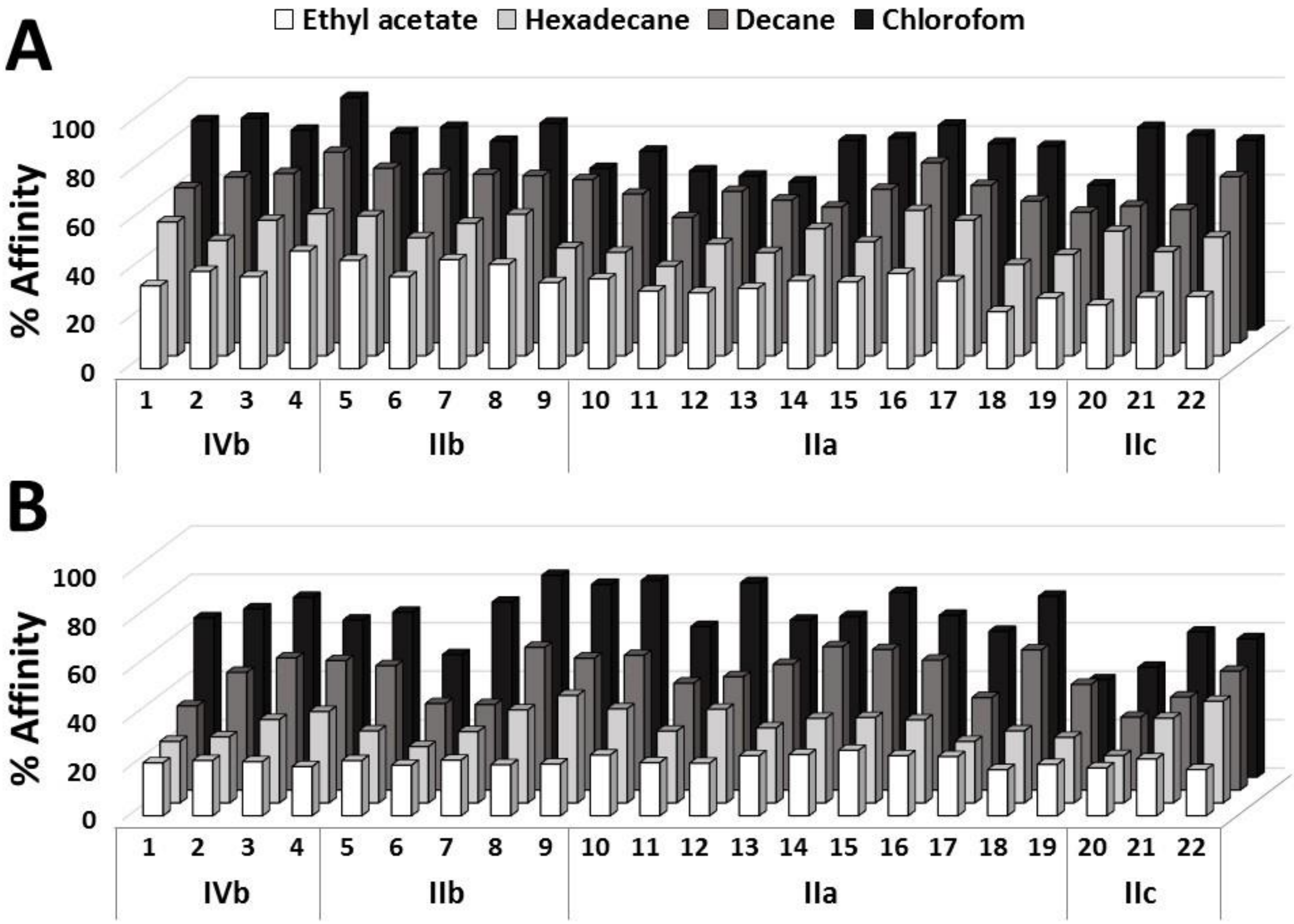

Supplementary Figure 3. Affinity (%) of 22 *L. monocytogenes* strains to solvents in stationary phase at 37°C (A) and 4°C (B). Data are shown as the mean of four measurements.
